# Hahnemann’s Writings and Rubrick

**Published:** 1888-03

**Authors:** M. W. Van Denburg


### HAHNEMANN'S WRITINGS AND RUBRICK.

In a very thoughtful paper (read before the New York State Homœopathic Society), on these subjects, Dr. M. W. Van Denburg considers how the mass of materia medica now collected can be made more available. He condemns, as we do, the chopped-up style of the so-called condensed works, where merely the husk is preserved and the kernel thrown out. While we do not altogether agree with Dr. Van Denburg's views, we are heartily glad to see such discussions of this subject. In concluding his paper, Dr. Van Denburg says:

"It is not incumbent upon us, as good and faithful disciples of Hahnemann, to copy his faults as well as his virtues. Neither are we called upon to regard him as inspired or infallible. He himself would be the first to repudiate such implications. It is rather our part to inquire, in the calmest scientific spirit, what is required of *Materia Medica Pura?*

"The first requirement is that it be truthful. The universality of the law of *similia* is not here under discussion, only materia medica. The second requirement is that it should be *available* for use with the *least possible* expenditure of time and labor.

"Efforts in this direction have been untiring. We have condensed and recondensed materia medicas; repertories large and small have consumed years of patient labor and helped in a very large degree to make up for deficiencies and confusion. Without them it is hard to see how we could practice medicine at all."

